# Supplementary material for: Splice-Junction-Based Mapping of Alternative Isoforms in the Human Proteome
Source: Cell Rep. Author manuscript; Available in PMC 2020 Jan 15. (PMC6961840; doi:10.1016/j.celrep.2019.11.026)
Supplement: 3 [file NIHMS1546469-supplement-3.zip › DF2/PXD0010154/Testis-70-O00468-VLGESPVPHTVLNLKEPLYVGGAPDFSK.pdf]

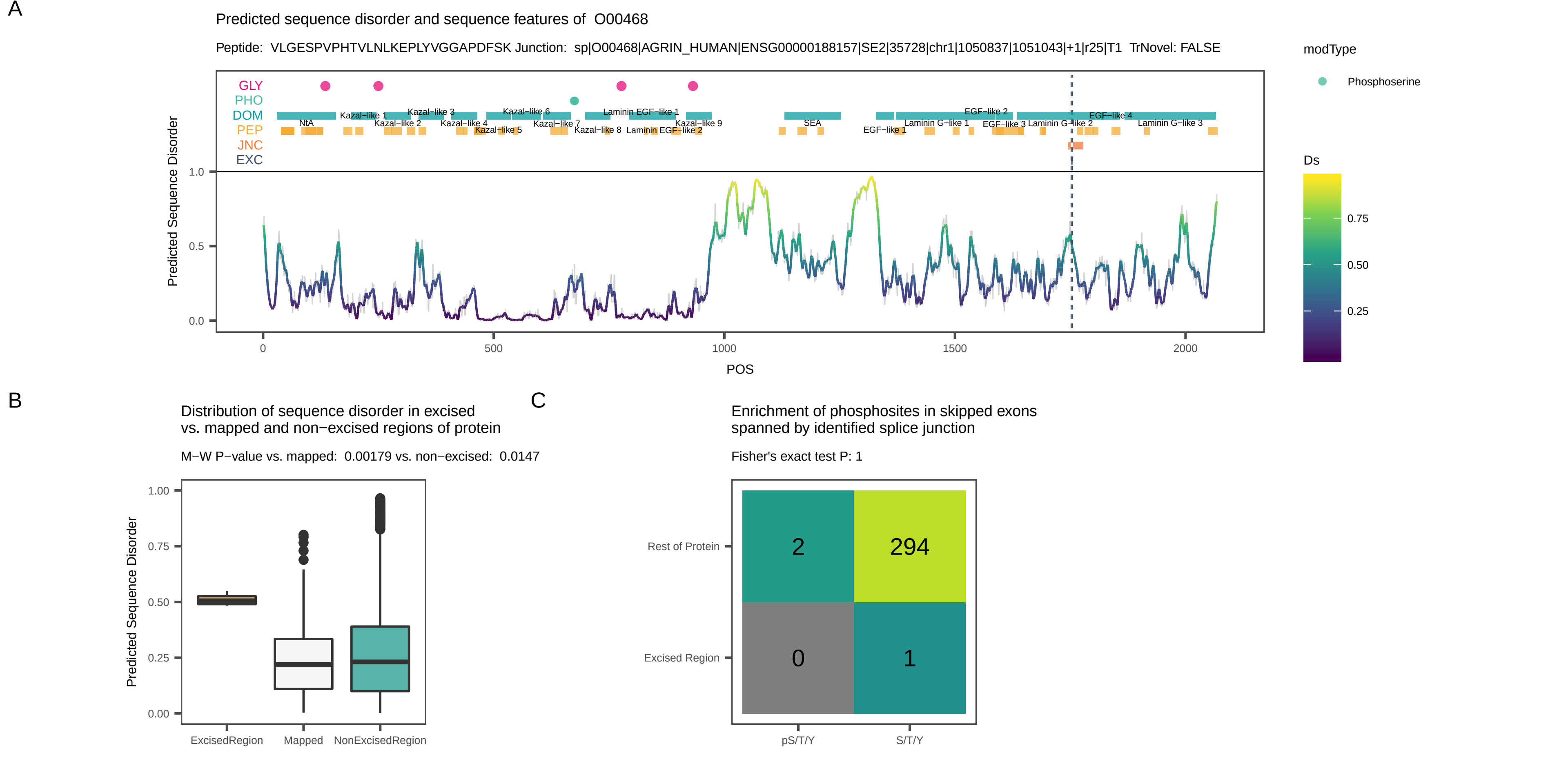
A
Predicted sequence disorder and sequence features of O00468
Peptide: VLGESPVPHTVLNLKEPLYVGGAPDFSK Junction: sp|O00468|AGRIN_HUMAN|ENSG00000188157|SE2|35728|chr1|1050837|1051043|+1|r25|T1 TrNovel: FALSE
modType
Phosphoserine
GLY
PHO
DOM
PEP
JNC
EXC
Ds
0.75
0.50
0.25
Predicted Sequence Disorder
POS
B
Distribution of sequence disorder in excised vs. mapped and non−excised regions of protein
M−W P−value vs. mapped: 0.00179 vs. non−excised: 0.0147
Predicted Sequence Disorder
ExcisedRegion
Mapped
NonExcisedRegion
C
Enrichment of phosphosites in skipped exons spanned by identified splice junction
Fisher's exact test P: 1
Rest of Protein
Excised Region
2
294
0
1
pS/T/Y
S/T/Y
